# Temporal Courses in EEG Theta and Alpha Activity in the Dynamic Health Qigong Techniques Wu Qin Xi and Liu Zi Jue

**DOI:** 10.3389/fpsyg.2017.02291

**Published:** 2018-01-08

**Authors:** Diana Henz, Wolfgang I. Schöllhorn

**Affiliations:** Institute of Sport Science, University of Mainz, Mainz, Germany

**Keywords:** EEG, health Qigong, dynamic Qigong, theta activity, alpha activity

## Abstract

Health Qigong is a common technique of Traditional Chinese Medicine applied to strengthen mental and physical health. Several studies report increases in EEG theta and alpha activity after meditative Qigong techniques indicating a relaxed state of mind. To date, little is known on the effects of dynamic Health Qigong techniques that comprise bodily movements on brain activity. In the current study, we compared effects of two dynamic Health Qigong techniques on EEG brain activity. Subjects performed the techniques *Wu Qin Xi* (five animals play) and *Liu Zi Jue* (six healing sounds) in a within-subjects design. Eyes-open and eyes-closed resting EEG was recorded before and immediately after each 15-min practice block. Additionally, the Profile of Mood States (POMS) questionnaire was administered at pretest, and after each 15-min practice block. Results show a decrease in alpha activity after 15 min, followed by an increase after 30 min in the Health Qigong technique *Liu Zi Jue*. Theta activity was decreased after 15 min, followed by an increase after 30 min in the technique *Wu Qin Xi*. Results of the POMS indicated an increased vigor-activity level with decreased fatigue and tension-anxiety levels in both techniques after 30 min of practice. Our results demonstrate different temporal dynamics in EEG theta and alpha activity for the Health Qigong techniques *Wu Qin Xi* and *Liu Zi Jue*. We hypothesize that the found brain activation patterns result from different attentional focusing styles and breathing techniques performed during the investigated Health Qigong techniques.

## Introduction

Health Qigong is a technique of Traditional Chinese Medicine (TCM) applied commonly to strengthen physical and mental health. In several studies on meditative Qigong practice beneficial effects on health have been observed (for an overview see Ng and Tsang, 2009). Effects of Qigong practice on electrocardiographic parameters (Lee et al., 2000, 2003), blood pressure (Lee et al., 2003; Cheung et al., 2005), and breathing frequency (Sun, 1988) were investigated. Positive effects of Qigong practice on mental health were shown in anxiety disorders (Lee et al., 2004; Abbott and Lavretsky, 2013; Chan et al., 2013), posttraumatic stress disorders (Grodin et al., 2008; Kim et al., 2013), major depression (Tsang et al., 2003; Wang C. W. et al., 2013; Wang F. et al., 2013; Yeung et al., 2013; Yin and Dishman, 2014; Liu et al., 2015; Martínez et al., 2015), in the burnout syndrome (Stenlund et al., 2009, 2012), and in tinnitus (Biesinger et al., 2010). In recent studies, a stress alleviating effect was shown in healthy practitioners (Posadzki et al., 2010; Terjestam et al., 2010; Glei et al., 2012; Sousa et al., 2012; Hwang et al., 2013; Shim, 2014; Wang et al., 2014).

An essential research question is how the beneficial effects of Qigong practice on physical and mental health are mediated by neurophysiological processes. Several studies using electroencephalography (EEG) and fMRI demonstrated changes of brain activity induced by Qigong meditation. Most studies report increases in theta and alpha oscillations after Qigong meditation as a correlate for a relaxed and attentive mind. The first studies on the effect of Qigong meditation on electrical brain activity reported alpha activity predominantly in the anterior brain regions (Wallace, 1970). More differentiated results of effects of meditational Qigong techniques on EEG activity are shown dependent on expertise level. Several studies demonstrated shifts in alpha activation from posterior to anterior regions during Qigong meditation (Zhang et al., 1988a,b; Jang et al., 2004; Qin et al., 2009). Yang et al. (1994) report effects of Zhanzhuang Qigong on brain activity. After 1 year of practice, the alpha index of the right frontal and right temporal regions increased significantly. The beta activity of the right frontal and right temporal regions decreased significantly. A synchronization of brain activity was observed. The effects were not observed after half a year of continuing meditation. Thus, it was a gradually adjusting process.

Psychophysiological states of wakefulness and arousal as measured in terms of activation of particular EEG frequency bands are commonly related to distinct self-reported experiences in regular Qigong meditators. Mostly, an increase of alpha activity is related to an experience of relaxation and well-being, whereas an increase of EEG theta activity is correlated to a self-report of mindfulness. One of the main aims in Buddhist meditation traditions is to reach the state of mindfulness, which is defined as an attentive state of mind (for an overview see Tomasino et al., 2014).

For instance, Faber et al. (2012) demonstrated EEG alpha-2 activity in posterior right parietal Brodmann areas 5, 7, 31, and 40 during Qigong meditation. It is argued that the found patterns of brain activation reflect self-reference, attention, and input-centered processing in Qigong meditation. Lee et al. (1997) investigated effects of ChunDoSunBup Qi-training on brain activity. The Qi-training comprises sound exercises, bodily motion, and meditation. Increases in alpha activity in ChunDoSunBup Qi-training were shown in the occipital regions in eyes-open conditions. The increase in occipital alpha activity was correlated to less self-reported state anxiety. One line of argumentation is that in ChunDoSunBup Qi-training activity of the occipital cortex is reduced and the thalamus is influenced. In a study by Pan et al. (1994) frontal mid-line theta rhythm during the concentrative Qigong state compared to the state of mind reached by non-concentrative Qigong engagement was demonstrated. Shim (2012) reported theta activity centering around the frontal lobe parts in Qigong masters and decreased alpha activity compared to beginners. The authors argue that Qigong experts maintained more deeply internalized and relaxed theta activity in the frontal lobe which reflects an attentive state of mind. Qigong masters show efficiency in keeping a relaxed and attentive mind around central midline during meditation (see also Tei et al., 2006a,b, 2009).

On a more structural level, Lehmann et al. (2012) showed reduced functional connectivity between cortical sources in Qigong meditation with reduced functional interdependence between brain regions. The authors argue that the reported subjective experience of non-involvement, detachment, and letting go, as well as of all-oneness and dissolution of ego borders during meditation is mirrored in the found pattern of brain activity.

Cheng et al. (2010) demonstrated an effect of Qigong meditation on prefrontal activation. Practitioners showed in comparison to non-practitioners of Qigong meditation a significant decrease in deoxyhemoglobin levels suggesting an increase in prefrontal activation during Qigong meditation.

Two fMRI studies report changes in brain function under the state of Qigong during pain exposure in Qigong masters (Chan et al., 2006; Yu et al., 2007). Functional activation in the SII-insula region and other brain areas was reported, whereas a functional suppression under the state of Qigong meditation was observed. It is argued that the found functional suppression in brain regions may be responsible for the reduced pain sensation in Qigong masters under the Qigong state.

In summary, systematical effects of static Qigong meditation on EEG brain activity are demonstrated with most studies reporting increases in frontal theta and posterior alpha activity as a correlate for a relaxed and attentive mind.

Qigong comprises different techniques that are commonly divided into static and dynamic forms. Static forms comprise meditational techniques whereas dynamic forms afford bodily movements as a tool to direct practitioners' attention to reach a meditative state (Tsang et al., 2002). To our knowledge, there are no systematical studies on effects on brain activity of dynamic Qigong techniques that afford bodily movement. In an experimental study conducted in our working group, increases in theta and alpha activity over the whole scalp after physical training of the Qigong technique *Wu Qin Xi* were observed (Henz and Schöllhorn, 2017). The same pattern of results was replicated in two consecutive studies on the effects on brain activity in the Qigong technique *Wu Qin Xi* (Henz et al., 2014, 2015). From a qualitative point of view, the found brain activation patterns were in line with findings of studies conducted with static meditational Qigong. As in studies on static meditational Qigong, increased fronto-central theta, and posterior alpha activity was observed.

The main aim of the present study is to investigate acute effects on EEG brain activity in the dynamic Health Qigong techniques *Liu Zi Jue* (six healing sounds) and *Wu Qin Xi* (five animals play) in a comparative experimental design. The Health Qigong technique *Liu Zi Jue* is the art of expiration in producing six different sounds [xu (嘘), he (呵), hu (呼), si (呬), chui (吹), xi (嘻)]. Each sound is performed within a specific movement routine without moving the feet, with each sound and movement routine repeated six times. Inverse abdominal respiration is required while performing the practice, as well as producing the six sounds respectively during exhaling. While inhaling with lips closed and tongue reaching the palate, one should breath naturally through the nose and make the abdomen uplifted (Chinese Health Qigong Association, 2007). According to TCM theory moving lips and teeth with different forces can affect different organs and the circulation of Qi and blood in the vessels and in the meridians, this results in training of the organs, harmonizing the Qi and blood, and balancing the Yin and Yang (Jiang and Zou, 2013). Its diaphragmatic breathing may produce increased asynchronous and paradoxical breathing movements, and its prolonged expiration and slowing of the breathing rate is widely used and produces a satisfactory effect (for an overview see Yang and Wu, 2011). The Health Qigong technique *Wu Qin Xi* comprises a consecutive sequence of complex movement configurations. These configurations are the game of the five animals (tiger, deer, bear, monkey, bird) with each movement sequence performed between two and three times for several minutes. Practitioners are requested to focus on breathing when performing the movement sequences. According to theoretical assumptions of TCM *Wu Qin Xi* is an intervention to strengthen especially physical health in general (for an overview see Yang and Wu, 2011).

Only a few studies have been conducted to investigate the effects of *Liu Zi Jue* and *Wu Qin Xi* on physical and mental health. Studies on the dynamic Qigong technique *Liu Zi Jue* report beneficial effects on chronic obstructive pulmonary disease (Xiao and Zhuang, 2015), on pain consciousness, and on depression (Dong and Lee, 2013). Positive effects of *Wu Qin Xi* training are reported on lumbar spinal disease (Yeom et al., 2013; You et al., 2013, 2014; Zhang et al., 2014), blood lipid levels and the antioxidant enzyme activities (Chen, 2011).

To date, there are no systematical studies reported on the effects of *Liu Zi Jue* Qigong training on EEG brain activity that examine the underlying neuronal processes that mediate the positive effects on physical health. Recently, it was shown that *Liu Zi Jue* training made beneficial effects on pain consciousness and depression of elderly single women (Dong and Lee, 2013). As depression is correlated with reduced EEG alpha activity (see Başar et al., 2012), one hypothetical assumption could be derived that *Liu Zi Jue* might have a modulating effect on EEG brain activity which is one neurophysiological substrate for a subjective experience of increased well-being.

A second aim of the present study is to investigate the temporal course of EEG brain activity in both Qigong techniques. To our knowledge there are no systematical studies on the temporal course of EEG brain activity in the dynamic Qigong techniques *Wu Qin Xi* and *Liu Zi Jue*. One important question considering the design of interventions to achieve an optimum effect is the acute duration of Qigong practice. Does the theta and alpha activity increase with increasing practice duration? A further important research question is whether differences in the temporal course of EEG brain activity occur in the dynamic Qigong techniques *Wu Qin Xi* and *Liu Zi Jue*. In previous studies on Qigong meditation modulations of brain activity were observed already after a short-term practice of 10 (e.g., Faber et al., 2012) to 15 min (Lavallee et al., 2011; Shim, 2012).

From previous studies on Qigong meditation, we suppose that practicing the Qigong techniques *Wu Qin Xi* and *Liu Zi Jue* result in increased EEG theta and alpha activity. According to the theoretical framework of TCM, we hypothesize that practicing the Qigong technique *Liu Zi Jue* leads to stronger increases in EEG theta activity as a correlate for a concentrative meditational state compared to practice of the Qigong technique *Wu Qin Xi*. From a physiological perspective, we argue that in *Liu Zi Jue* the role of breathing is more reinforced due to producing the six sounds when performing the movements. Therefore, breathing behavior tends to be more regulated by adapting to the sound production than in *Wu Qin Xi*. Recent EEG studies have shown that abdominal breathing techniques lead to increased frontal theta activity (e.g., Yu et al., 2011; Chervin et al., 2012; Park and Park, 2012). Considering breathing as a meditation technique, it was observed that Shaolin Dan Tian Breathing increases EEG frontal theta activity (Chan et al., 2011). The authors argue that the observed increase in frontal theta activity in Shaolin Dan Tian Breathing is a correlate for an attentive mind.

Another line of argumentation for a stronger increase in EEG theta activity in *Liu Zi Jue* than in *Wu Qin Xi* is that attentional processes are more guided by directing the focus of attention on breathing due to sound production than on mere movement performance which might lead to stronger internalized attention reflecting in increased frontal theta activity. Van der Lubbe et al. (2014) showed that the focus of spatial attention leads to changes in EEG brain activity. Additionally, movement configurations in *Liu Zi Jue* are reported from practitioners to be less complex than in *Wu Qin Xi*. Therefore, practitioners possibly tend to spend more effort on control of breathing than on control of complex movement configurations as in *Wu Qin Xi*.

From this line of argumentation and on basis of previous studies on the effect of abdominal breathing techniques, we expect a stronger increase in frontal EEG theta activity in *Liu Zi Jue* than in *Wu Qin Xi*. From results of previous studies on Qigong meditation and *Wu Qin Xi* (Henz and Schöllhorn, 2017; Henz et al., 2014, 2015) we hypothesize effects in *Liu Zi Jue* and *Wu Qin Xi* after 15 min with increases in theta activity in *Liu Zi Jue* after 30 min compared to *Wu Qin Xi*.

The aims of the present study are:

1. The analysis of the spontaneous eyes-closed and eyes-open EEG spontaneous activity before and after training of the dynamic Qigong techniques *Wu Qin Xi* and *Liu Zi Jue*.
2. Comparison of the temporal course of EEG brain activity after a 15-, and a 30-min practice of the Qigong techniques *Wu Qin Xi* and *Liu Zi Jue*.

## Methods

### Participants

Twenty subjects (mean age 32.8 years, age range 21–50, 8 males, 12 females) volunteered in this study. Subjects were recruited from the Qigong workshops at the Institute of Sports Science of the University of Mainz and from sports science courses and had at least regular Qigong training experience of 1 year. The subjects were all healthy, and had no current diseases or a history of neurological impairments or intake of medication that may have affected EEG recordings. All subjects were naïve as to the purpose of the current study. All subjects gave written informed consent. The experimental procedures were approved by the local ethics committee at the Johannes Gutenberg University of Mainz, Germany. All experimental procedures were carried out in accordance with the Declaration of Helsinki.

### Experimental procedure

The subjects were sat comfortably in a dimly-lit isolated room. At each measurement time point, participants began with a resting condition. Spontaneous EEG of the subject was recorded for 2 min for eyes-open, and 2 min for eyes-closed conditions. Then, subjects were required to perform two consecutive 15-min Qigong practice sessions. The experiment contained two tasks: Participants were required to perform the dynamic Qigong technique *Liu Zi Jue* and *Wu Qin Xi* in a within-subjects design at 2 consecutive days. The Qigong technique *Liu Zi Jue* consisted of six distinct movement routines, with each movement routine repeated six times. In the Qigong technique *Wu Qin Xi*, participants performed the five movement sequences tiger, deer, bear, monkey, and bird. Participants were required to coordinate their breathing with the prescribed movements in both Qigong conditions. Each of the both Qigong techniques was performed on a separate day. Experimental conditions were randomized. All training sessions, were performed with eyes-open. EEG data were obtained during the six resting conditions: (1) pretraining (*Wu Qin Xi*) rest, (2) pretraining (*Liu Zi Jue*) rest, (3) post-Qigong (*Wu Qin Xi*) practice after 15 min, (4) post-Qigong (*Wu Qin Xi*) practice after 30 min, (5) post-Qigong (*Liu Zi Jue*) practice after 15 min, (6), post-Qigong (*Liu Zi Jue*) practice after 30 min, which were then used for subsequent analyses.

To assess the psychological mood, the Profile of Mood States (POMS) questionnaire (McNair et al., 1971) was submitted to the subjects before the experiment, and after each experimental task. The assessment of mood was focused on vigor-activity, fatigue-inertia, and tension-anxiety.

### EEG recording details

Electroencephalography (EEG) was recorded by using the Micromed Brainquick amplifier and Micromed Brainspy software (Micromed, Venice, Italy). Recordings were made from Fp1, Fp2, F3, F7, Fz, F4, F8, C3, Cz, C4, T3, T4, P3, P7, Pz, P4, P8, O1, O2 placed according to the international 10–20 system with reference to the nose. All electrode impedances were kept at 10 kΩ or below. The EEG signals were continuously recorded and digitized at a sampling rate of 256 Hz. The EEG signal was amplified with a fixed time constant of 0.3 s with a high-pass filter at 0.5 Hz, and a low-pass filter at 120 Hz (frequency range: 0.5–120 Hz). Electrooculography (EOG) was monitored placed at the medial upper and lateral orbital rim of the right eye (time constant: 0.3 s; high pass filter: 0.1 Hz; low pass filter: 120 Hz; frequency range: 0.5–120 Hz).

### EEG analysis

#### Spontaneous EEG analysis

The spontaneous EEG was recorded before and after the Qigong interventions for 2 min with eyes-closed, and 2 min eyes-open conditions. Subsequent analyses were performed separately for eyes-closed and eyes-open conditions. The EEG and EOG signals were visually evaluated and portions of the data that contained aberrant eye movements, muscle movements of artifacts were removed. The EEG was analyzed and Discrete Fast Fourier Transform was used to obtain the mean power amplitudes in theta (4–7.5 Hz), low-frequency alpha-1 (8–10 Hz), high-frequency alpha-2 (10–12.5 Hz), beta (13–29.5 Hz), and gamma (30–40 Hz) bands. The ranges of high- and low-frequency alpha bands were defined according to previous studies by Aeschbach et al. (1999), and Cantero et al. (2002).

### Statistical analysis

A statistical comparison of power of theta, alpha-1, alpha-2, beta, and gamma bands was done by repeated-measure analyses of variance (ANOVA) including the within-subject factors as Qigong technique (*Wu Qin Xi, Liu Zi Jue*), time (pretest, post-Qigong 15 min, post-Qigong 30 min), experimental condition (eyes-open, eyes-closed), and location (Frontal, Central, Temporal, Parietal, Occipital). Additionally, partial eta-squared (η_*p*_^2^) was calculated to determine effect sizes. ANOVAs were followed by Bonferroni corrected *post-hoc* tests for further comparisons. POMS data were subjected to repeated-measure ANOVAs including the within-subject factors as Qigong technique (*Wu Qin Xi, Liu Zi Jue*), and time (pretest, post-Qigong 15 min, post-Qigong 30 min). Additionally, partial eta-squared (η_*p*_^2^) was calculated to determine effect sizes. Subsequently, Bonferroni corrected *post-hoc* tests were calculated for further comparisons. Effects were considered to be statistically significant when the *p*-values were less than 0.05.

## Results

### Statistical description: spontaneous EEG

**Figure 1:**
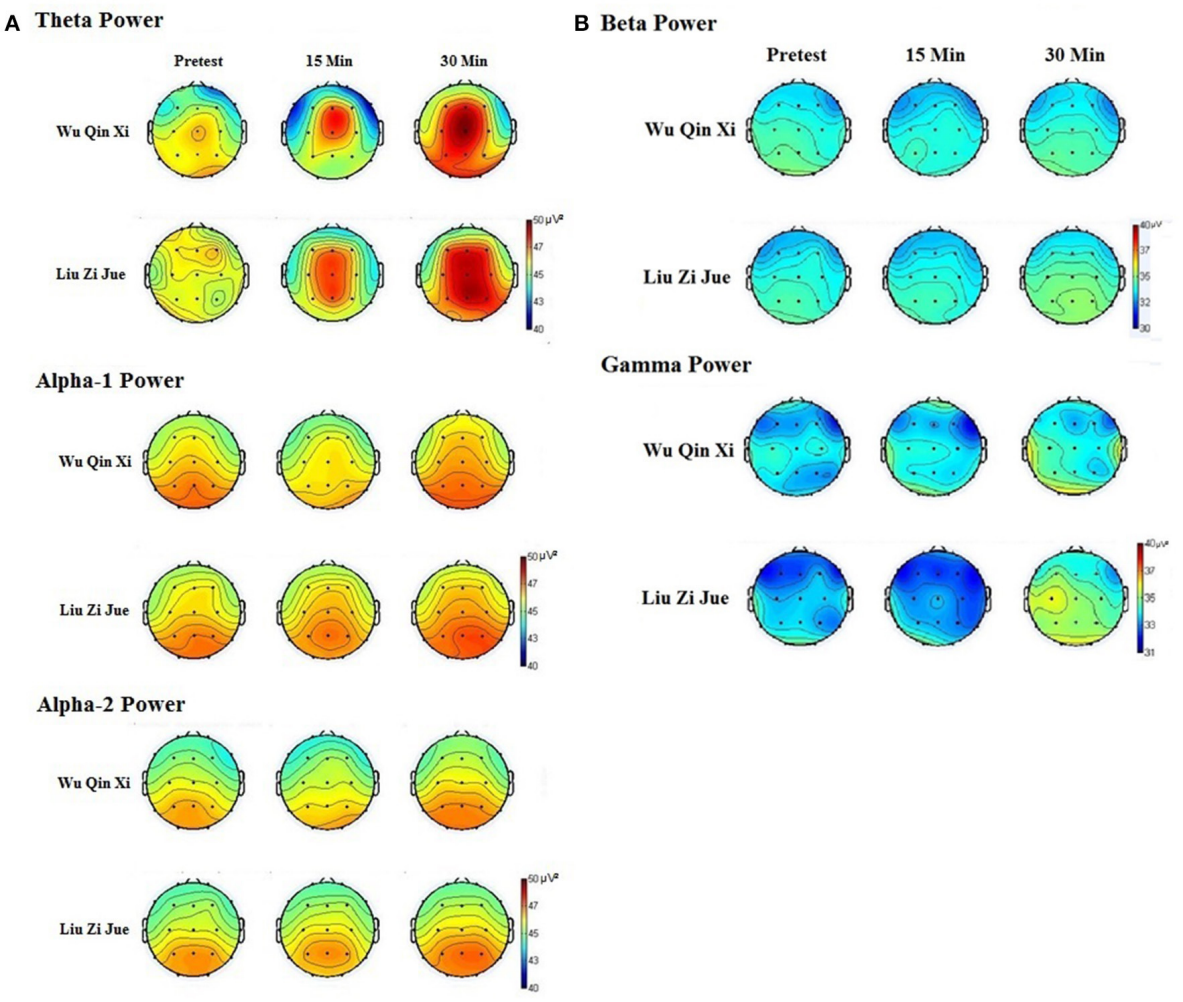
**(A)** Spontaneous EEG theta, alpha-1, and alpha-2 activity at resting baseline, and after 15 and 30 min Qigong practice. Results show different temporal courses in EEG theta, alpha-1, and alpha-2 activity in the dynamic Health Qigong techniques *Wu Qin Xi* and *Liu Zi Jue* during practice. **(B)** Spontaneous EEG beta and gamma activity at resting baseline, and after 15 and 30 min Qigong practice. Results show different temporal courses in EEG gamma activity in the dynamic Health Qigong techniques *Wu Qin Xi* and *Liu Zi Jue* during practice.

Figures 1A,B show the mean power spectra for the theta, alpha-1, alpha-2, beta, and gamma band in *Liu Zi Jue* and *Wu Qin Xi*. Main statistical outcomes for the EEG data are presented in Table 1. The ANOVA of theta responses revealed highly significant differences for Qigong technique, *F*_(1, 19)_ = 9.20, *p* = 0.007, η_*p*_^2^ = 0.33. *Post-hoc* comparisons showed that the spontaneous EEG theta power was significantly higher in *Liu Zi Jue* than in *Wu Qin Xi, p* = 0.009. The ANOVA of theta responses revealed significant differences for time, *F*_(2, 38)_ = 4.16, *p* = 0.023, η_*p*_^2^ = 0.18. *Post-hoc* comparisons showed that the spontaneous EEG theta power was significantly higher after 30 min, than after 15 min, *p* = 0.028, and compared to the resting baseline, *p* = 0.015. The ANOVA of theta responses revealed significant differences between locations, *F*_(4, 76)_ = 3.304, *p* = 0.015, η_*p*_^2^ = 0.14. *Post-hoc* comparisons showed that spontaneous EEG theta power at frontal, and central electrodes was higher than that of temporal, *p* < 0.05 each, parietal, *p* < 0.05 each, and occipital electrodes, *p* < 0.05 each. Subsequent analyses showed that theta power was most increased in the frontal region at the electrode Fz, *p* < 0.05, and in the central region at electrode Cz, *p* < 0.05. The ANOVA of theta responses revealed significant results for technique × time, *F*_(2, 38)_ = 4.72, *p* = 0.020, η_*p*_^2^ = 0.18. *Post-hoc* comparisons showed that in *Wu Qin Xi* theta power was significantly higher after 30 min than in *Liu Zi Jue, p* = 0.01. The ANOVA of theta responses revealed significant effects for technique x location, *F*_(4, 76)_ = 3.029, *p* = 0.023, η_*p*_^2^ = 0.14. *Post-hoc* comparisons showed increased theta power in *Liu Zi Jue* in parietal locations, compared to *Wu Qin Xi, p* = 0.016. The ANOVA of theta responses revealed significant effects for time × location, *F*_(8, 152)_ = 2.39, *p* = 0.027, η_*p*_^2^ = 0.11.

**Table 1:** *P*-values and partial eta-squared (η_*p*_^2^) for the EEG data for the factors Qigong technique, time, location, and the technique × time, technique × location, and time × location interactions, Statistical values are depicted for the theta, alpha-1, alpha-2, beta, and gamma band.

	**Qigong technique**		**Time**		**Location**		**Technique × Time**		**Technique × Location**		**Time × Technique**	
	***p***	**η*_*p*_*^2^**	***p***	**η*_*p*_*^2^**	***p***	**η*_*p*_*^2^**	***p***	**η*_*p*_*^2^**	***p***	**η*_*p*_*^2^**	***p***	**η*_*p*_*^2^**
Theta	0.007	0.33	0.023	0.18	0.015	0.25	0.020	0.18	0.023	0.14	0.027	0.11
Alpha-1	0.013	0.28	0.011	0.29	0.025	0.14	0.037	0.16	n.s.	–	n.s.	–
Alpha-2	0.017	0.20	0.016	0.23	0.032	0.11	0.028	0.13	n.s.	–	n.s.	–
Beta	n.s.	–	n.s.	–	n.s.	–	n.s.	–	n.s.	–	n.s.	–
Gamma	0.032	0.11	0.018	0.19	0.045	0.08	0.013	0.28	n.s.	–	n.s.	–

The ANOVA of alpha-1 responses revealed significant differences for Qigong technique, *F*_(1, 19)_ = 7.45, *p* = 0.013, η_*p*_^2^ = 0.28. *Post-hoc* comparisons showed that the spontaneous EEG alpha-1 power was higher in *Liu Zi Jue*, than in *Wu Qin Xi, p* = 0.017. The ANOVA of alpha-1 responses revealed significant differences for time, *F*_(2, 38)_ = 4.86, *p* = 0.011, η_*p*_^2^ = 0.29. *Post-hoc* comparisons showed that the spontaneous EEG alpha-1 power was significantly higher after 30 min, than after 15 min, *p* = 0.021, and compared to the resting baseline, *p* = 0.011. There was no difference between resting baseline, and 15 min. The ANOVA of alpha-1 responses revealed significant results for Qigong technique x time, *F*_(2, 38)_ = 3.612, *p* = 0.037, η_*p*_^2^ = 0.16. *Post-hoc* comparisons showed that in *Wu Qin Xi* alpha-1 power was significantly decreased after 15 min compared to 30 min, *p* = 0.045, and baseline rest, *p* = 0.014. In *Liu Zi Jue*, alpha-1 activity was increased after 15 min compared to baseline, *p* = 0.027, and after 30 min, *p* = 0.034. The ANOVA of alpha-1 responses revealed significant differences between locations, *F*_(4, 76)_ = 2.958, *p* = 0.025, η_*p*_^2^ = 0.14. *Post-hoc* comparisons showed that spontaneous EEG alpha-1 power was higher at central, parietal, and occipital electrodes than that of frontal, and temporal electrodes, *p* < 0.05 each. Subsequent analyses showed that alpha-1 power was most increased in the frontal region at the electrodes Fz, *p* < 0.05, and F4, *p* < 0.05.

The ANOVA of alpha-2 responses revealed significant differences for Qigong technique, *F*_(1, 19)_ = 6.85, *p* = 0.017, η_*p*_^2^ = 0.20. *Post-hoc* comparisons showed that the spontaneous EEG alpha-2 power was significantly higher in *Liu Zi Jue*, than in *Wu Qin Xi, p* = 0.05. The ANOVA of alpha-2 responses revealed significant differences for time, *F*_(2, 38)_ = 4.66, *p* = 0.016, η_*p*_^2^ = 0.23. *Post-hoc* comparisons showed that the spontaneous EEG alpha-2 power was significantly higher after 30 min, than after 15 min, *p* = 0.025, and compared to the resting baseline, *p* = 0.01. The ANOVA of alpha-2 responses revealed significant results for technique x time, *F*_(2, 38)_ = 3.92, *p* = 0.028, η_*p*_^2^ = 0.13. *Post-hoc* comparisons showed that in *Wu Qin Xi* EEG alpha-2 power was decreased after 15 min compared to 30 min, *p* = 0.035, and after resting baseline, *p* = 0.018. In *Liu Zi Jue* alpha-2 power was increased after 30 min compared to 15 min, *p* = 0.041, and resting baseline, *p* = 0.031. There was no significant difference between 15 min, and resting baseline. The ANOVA of alpha-2 responses revealed significant differences between locations, *F*_(4, 76)_ = 2.799, *p* = 0.032, η_*p*_^2^ = 0.11. *Post-hoc* comparisons showed that spontaneous EEG alpha-2 power was higher at parietal and occipital electrodes, than that of frontal, central, and temporal electrodes, *p* < 0.05 each. Subsequent analyses showed that alpha-2 power was most increased in the parietal region at the electrode P4, *p* < 0.05, and in the occipital region at electrode O2, *p* < 0.05.

The ANOVA of beta responses revealed no significant differences for Qigong technique, time, experimental condition, neither for locations.

The ANOVA of gamma responses revealed significant differences for Qigong technique, *F*_(1, 19)_ = 5.33, *p* = 0.032, η_*p*_^2^ = 0.11. The ANOVA of gamma responses revealed significant differences for time, *F*_(2, 38)_ = 4.45, *p* = 0.018, η_*p*_^2^ = 0.19. *Post-hoc* comparisons showed that the spontaneous EEG gamma power was significantly higher after 30 min, than after 15 min, *p* = 0.038, and compared to the resting baseline, *p* = 0.011. The ANOVA of gamma responses revealed significant results for technique x time, *F*_(2, 38)_ = 4.92, *p* = 0.013, η_*p*_^2^ = 0.28. *Post-hoc* comparisons showed increased gamma power in *Liu Zi Jue* after 30 min compared to *Wu Qin Xi* at each measurement point, *p* = 0.019. Further, in *Liu Zi Jue* gamma power was significantly higher after 30 min, than after 15 min, *p* = 0.021, and compared to baseline rest, *p* = 0.009. There was no difference in gamma activity between baseline rest and after 15 min in *Liu Zi Jue*. The ANOVA of gamma responses revealed significant differences between locations, *F*_(4, 76)_ = 2.56, *p* = 0.045, η_*p*_^2^ = 0.08. *Post-hoc* comparisons showed that spontaneous EEG gamma power at central electrodes was higher than that of frontal, parietal, temporal, and occipital electrodes, *p* < 0.05 each.

### POMS questionnaire

**Figure 2:**
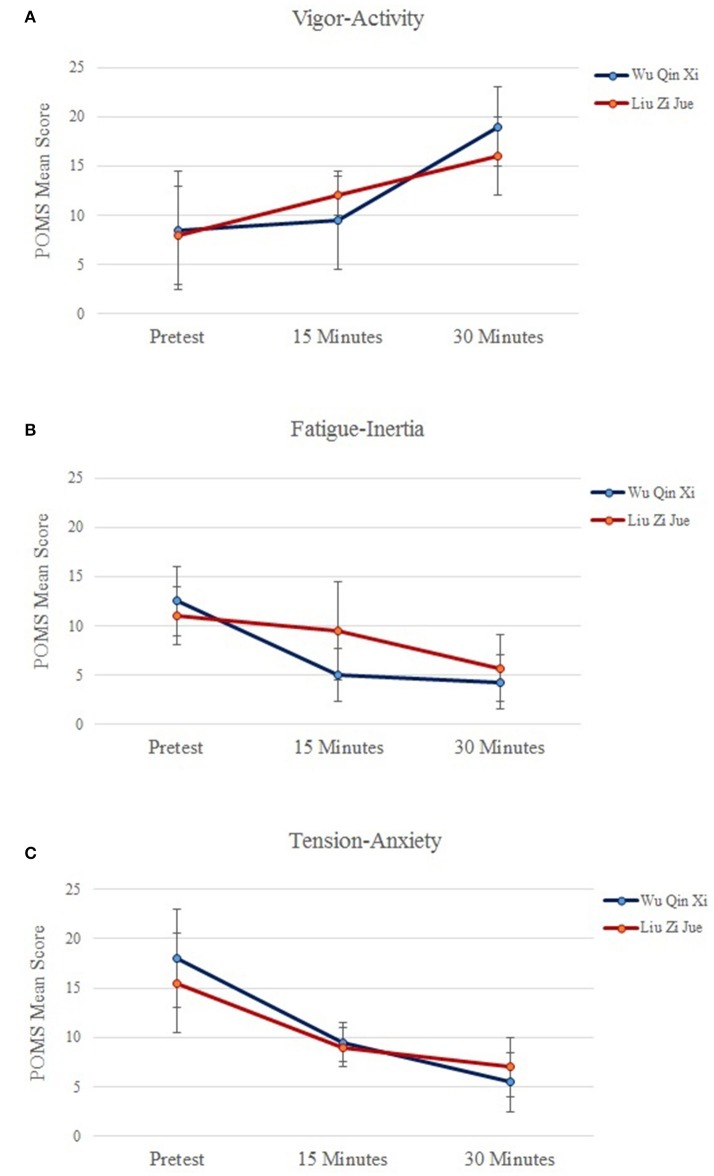
**(A–C)** Means and SDs of the POMS scales vigor-activity, fatigue-inertia, and tension-anxiety in the dynamic Health Qigong techniques *Wu Qin Xi* and *Liu Zi Jue* at pretest, after 15 min, and after 30 min of practice. Results show increased vigor-activity levels with decreased fatigue and tension-anxiety levels in both Health Qigong techniques after 30 min of practice.

Means and standard deviations of the POMS scales vigor-activity, fatigue-inertia, and tension-anxiety are depicted in Figure 2. Main statistical outcomes for the POMS scales are presented in Table 2. The ANOVA for the scale vigor-activity revealed significant differences for the factor Qigong technique, *F*_(1, 19)_ = 5.46, *p* = 0.022, η_*p*_^2^ = 0.18, with a higher vigor-activity level in *Wu Qin Xi* than in *Liu Zi Jue, p* = 0.02. The ANOVA for the factor time showed significant differences for training duration, *F*_(2, 38)_ = 3.97, *p* = 0.034, η_*p*_^2^ = 0.10. *Post-hoc* comparisons revealed that the vigor-activity level was significantly higher after 30 min of practice than at pretest, *p* = 0.02, and after 15 min of practice, *p* = 0.04. The technique × time interaction was significant, *F*_(2, 38)_ = 3.61, *p* = 0.042, η_*p*_^2^ = 0.07.

**Table 2:** *P*-values and partial eta-squared (η_*p*_^2^) for the data of the POMS-questionnaire for the factors Qigong technique, time, and the Qigong technique × time interaction for the scales vigor-activity, fatigue-inertia, and tension-anxiety.

	**Qigong technique**		**Time**		**Qigong technique × Time**	
	***p***	**η*_*p*_*^2^**	***p***	**η*_*p*_*^2^**	***p***	**η*_*p*_*^2^**
Vigor-activity	0.022	0.18	0.034	0.10	0.042	0.07
Fatigue-inertia	n.s.	–	0.028	0.14	0.040	0.08
Tension-anxiety	n.s.	–	0.021	0.12	n.s.	–

The ANOVA for the scale fatigue-inertia revealed no significant difference for the factor Qigong technique. The ANOVA for the factor time showed significant differences for training duration, *F*_(2, 38)_ = 4.58, *p* = 0.028, η_*p*_^2^ = 0.14. *Post-hoc* comparisons showed that the fatigue-inertia level was significantly reduced after 15 min, *p* = 0.02, and 30 min of practice, *p* = 0.02, compared to pretest. The technique x time interaction was significant, *F*_(2, 38)_ = 3.82, *p* = 0.040, η_*p*_^2^ = 0.08.

The ANOVA for the scale tension-anxiety revealed no significant effect for Qigong technique. A further ANOVA showed significant differences for training duration, *F*_(2, 38)_ = 4.20, *p* = 0.021, η_*p*_^2^ = 0.12. *Post-hoc* comparisons showed that the tension-anxiety level was decreased after 30 min of practice compared to pretest, *p* = 0.02, and 15-min practice, *p* = 0.04. The technique × time interaction was not significant.

## Discussion

The current literature includes several previous investigations on effects of static Qigong meditation on EEG brain activity. Most studies report an increase in EEG theta and alpha activity during and after static Qigong meditation. In the present study, we investigated effects of the dynamic Qigong techniques *Wu Qin Xi* and *Liu Zi Jue* with emphasis on the temporal course of brain activation at training durations of 15 and 30 min, respectively. We demonstrated an increase of midline fronto-central theta and posterior alpha-1, and alpha-2 activity after practice of the dynamic Qigong techniques *Wu Qin Xi* and *Liu Zi Jue*. Our results mirror the findings of previous studies of effects on EEG brain activity after Qigong meditation. From a qualitative point of view, a comparable effect for the dynamic Qigong techniques *Wu Qin Xi* and *Liu Zi Jue* on EEG brain activity as found in studies on static meditational Qigong can be observed. Dynamic *Wu Qin Xi* and *Liu Zi Jue* Qigong training induces a relaxed and attentive mind as indicated by an increase in midline fronto-central theta and posterior alpha-1, and alpha-2 activity after a practice duration of 30 min. Thus, a training schedule with two practice cycles of 15 min of the movement sequences in *Wu Qin Xi* and *Liu Zi Jue* is sufficient to approach the desired brain state. POMS data mirror the EEG findings with decreased fatigue and tension-anxiety scores in both techniques after 30 min of practice. We conclude that increases in EEG theta and alpha activity lead to a psychophysiological state of relaxation that reduces signs of fatigue and subjective sensations of tension and anxiety. Summarizing, a relaxing effect of the dynamic Qigong techniques *Wu Qin Xi* and *Liu Zi Jue* in sense of an evidence-based approach can be stated. Our results indicate that both dynamic Qigong techniques induce a centered and attentive state of mind indicated by increases in fronto-central midline theta activity. This psychophysiological brain state is clearly distinguishable from mind-wandering, i.e., thoughts are not remaining on the control of breathing and experience of bodily sensations but on task-unrelated contents. Empirical evidence shows that frontal EEG theta activity is activated in attentional processes and correlates negatively with the default mode network in resting state as it is activated in mind-wandering (Scheeringa et al., 2008). Summarizing, one main finding of the present study is that *Wu Qin Xi* and *Liu Zi Jue* induce comparable effects in EEG theta, alpha-1, and alpha-2 activity after 30 min. Therefore, a relaxing effect and fostering of an attentive state of mind can be stated (Hong and Cho, 2012; Chou and Tsai, 2014).

The finding of increased fronto-central midline theta activity, and increased posterior alpha activity as shown in previous studies on the dynamic Qigong technique *Wu Qin Xi* (Henz and Schöllhorn, 2017; Henz et al., 2015) was replicated in the current study. Similar activations of brain activity are obtained in *Liu Zi Jue* training. Our results are in line with previous studies on static meditational Qigong techniques. For instance, frontal mid-line theta rhythm during the concentrative Qigong state compared to the state of mind reached by non-concentrative Qigong meditation was shown by Pan et al. (1994). In the same manner, Shim (2012) demonstrated frontal theta activity after Qigong meditation in experienced practitioners. In a study conducted by Aftanas and Golocheikine (2001) EEG brain activity was examined in meditation in eyes-open and eyes-closed conditions. Depending on eyes-open and eyes-closed conditions, different patterns of anterior and midline theta activity occurred. The authors argue that the found theta activity reflects internalized attentional processes during meditation that are dependent on eyes-open and eyes-closed states. Considering EEG alpha activity, the findings are in line with previous studies that stated increases in EEG alpha activity in static meditational Qigong. According to previous studies (Zhang et al., 1988a,b; Yang et al., 1994; Jang et al., 2004; Qin et al., 2009; Faber et al., 2012), a shift of EEG activity from posterior to anterior regions was observed in *Wu Qin Xi* and *Liu Zi Jue* in the present study.

The highlighted finding of this study is that different temporal courses with different topographical patterns in spontaneous EEG brain activity in the frequency bands theta, alpha-1, alpha-2, and gamma are obtained in *Wu Qin Xi* and *Liu Zi Jue*. Considering the temporal course of EEG activity in the alpha-1 and alpha-2 band stronger fluctuations in *Wu Qin Xi* than in *Liu Zi Jue* are observed. In *Liu Zi Jue* continuous increases of alpha-1 in frontal regions, and alpha-2 activity in parietal regions is obtained after 15 and 30 min, respectively. In *Wu Qin Xi*, a decrease compared to baseline rest of EEG alpha-1 activity is obtained in central, and parietal regions after 15 min, followed by an increase after 30 min. A similar pattern of results is observed for alpha-2 activity. A significant decrease in alpha-2 activity is obtained after 15 min, followed by an increase after 30 min in parietal areas. Considering theta activity, we obtained increases in parietal areas after 15 min in *Liu Zi Jue* compared to *Wu Qin Xi* training after 15 min. In *Wu Qin Xi*, occipital theta activity was increased after 30 min, compared to 15 min, and baseline rest. Gamma activity was observed only in *Liu Zi Jue* after 30 min in left central regions. POMS data on the vigor-activity scale show a significant technique by time interaction with a stronger fluctuation of vigor-activity levels in *Wu Qin Xi* during practice. This finding on the subjective psychophysiological state reflects the results obtained in the EEG alpha-1 and alpha-2 bands.

In summary, stronger fluctuations regarding the temporal course of alpha-1 and alpha-2 activity in *Wu Qin Xi* compared to *Liu Zi Jue* are demonstrated. In *Liu Zi Jue*, EEG alpha-1 and alpha-2 activity increased more quickly after 15 min than in *Wu Qin Xi*. Theta activity increased in parietal regions in *Liu Zi Jue*. We discuss three lines of argumentation for the obtained results in the present study: (1) the role of enhanced breathing in *Liu Zi Jue*, (2) the role of cognitive and internalized attentional processes due to properties of the practice sequences, and (3) the interaction of attentional processes and motor control in both movement sequences.

From previous studies on Qigong meditation, we hypothesized that practicing the Qigong techniques *Wu Qin Xi* and *Liu Zi Jue* result in increases in frontal theta and posterior alpha activity. According to the theoretical framework of TCM, we hypothesized that practicing the Qigong technique *Liu Zi Jue* leads to stronger increases in EEG theta activity as a correlate for a concentrative meditational state compared to practice of the Qigong technique *Wu Qin Xi*. From a physiological perspective, we argue that in *Liu Zi Jue* the role of breathing is more reinforced due to producing the six sounds when performing the movements. Therefore, breathing behavior tends to be more regulated by adapting to the sound production than in *Wu Qin Xi*. Recent EEG studies have shown that abdominal breathing techniques lead to increased frontal theta activity (e.g., Yu et al., 2011; Chervin et al., 2012; Park and Park, 2012). Considering breathing as a meditation technique, it was observed that Shaolin Dan Tian Breathing increases EEG frontal theta activity (Chan et al., 2011). The authors argue that the observed increase in frontal theta activity in Shaolin Dan Tian Breathing is a correlate for an attentive mind. Further, several studies have shown that abdominal breathing enhances EEG alpha activity. For instance, Arambula et al. (2001) demonstrated increases in EEG alpha activity in abdominal breathing techniques. Increased alpha band activity with decreased theta band activity was obtained in abdominal breathing during Zen practice (Arita, 2012). Comparing alpha-1 and alpha-2 activity Fumoto et al. (2004) showed increases in alpha-1 activity with disappearance of alpha-2 activity in voluntary abdominal breathing. From these results we argue that the found pattern of activation in the alpha-1 and alpha-2 band with stronger increases after 15 min in *Liu Zi Jue* compared to *Wu Qin Xi*, reflect modulations of EEG brain activity due to the stronger reinforced attention on breathing in *Liu Zi Jue* than in *Wu Qin Xi*. The pattern of decreased EEG alpha-1 and alpha-2 activity after 15 min, and delayed increase after 30 min in *Wu Qin Xi* possibly mirrors differences in breathing behavior due to the practice structure with less control of breathing capacities by sound production as it is prescribed in *Liu Zi Jue*.

A second line of argumentation for a stronger increase in EEG theta activity in *Liu Zi Jue* than in *Wu Qin Xi* is that attentional processes are more guided by directing the focus of attention on breathing due to sound production than on mere movement performance. This might lead to stronger internalized attentional processing that mirrors in increased EEG frontal theta activity in *Liu Zi Jue*. In contrast, *Wu Qin Xi* affords less binding of breathing capacities. Further, movement sequences afford high capacities in spatial processing. This Qigong technique requires practitioners to move spatially. Spatial directions and distances are strongly determined which in consequence force practitioners to direct their attention at least partially to the external space to perform the movement sequences appropriately. We argue that the focus of attention during practicing the movement sequences mediates the effects on EEG brain activity. For instance, Van der Lubbe et al. (2014) showed that the focus of spatial attention leads to changes in EEG brain activity. From behavioral studies on the role of attentional performance it is known that an external focus of attention alleviates movement performance, and therefore requires less effort. For instance, it was shown that movement performance benefits from an external focus of attention in gymnastics (Abdollahipour et al., 2015). Several recent studies have provided evidence that movement efficiency is enhanced by an external focus (Zachry et al., 2005; Marchant et al., 2009). Benefits of directing attention to an external focus have been found to result in more effective motor performance than those inducing an internal focus by directing attention to the body movements themselves (Totsika and Wulf, 2003; Wulf et al., 2003, 2009; Wulf, 2007). It is argued that focusing on the intended movement effect facilitates the utilization of unconscious or automatic processes. This results in greater movement ease or fluidity (Wulf et al., 2001; Wulf and Lewthwaite, 2010). In contrast, focusing on one's own movements leads to a more conscious type of movement control, thereby constraining the motor system and disrupting automatic control processes (Wulf et al., 2001). It has been shown that relative to an internal focus, an external focus reduces attentional demands and results in the utilization of fast reflexive feedback loops (Wulf et al., 2001). Transferring these findings on the dynamic Qigong techniques examined in the current study, might explain the found differences in the temporal course of EEG theta and alpha-1 and alpha-2 activity in *Wu Qin Xi* and *Liu Zi Jue, Liu Zi Jue* induces a strongly internally directed attentional processing due to binding of breathing capacities on sound production. In contrast, *Wu Qin Xi* affords a strongly spatial processing due to moving in space to spatial directions, and due to its complex movement configurations.

From this line of argumentation, an important question arises that opens our third line of argumentation: does an internal focus of attention during dynamic Health Qigong practice lead to a more demanding type of movement control, and therefore binds more attention which finally results in increased EEG frontal theta activity? Especially in novice practitioners, an attentionally demanding motor learning process during Health Qigong practice could result in enhanced stress reduction mirrored by changes in EEG brain activity. We argue that one underlying cognitive mechanism is a working memory load which results from increased motor affordances during dynamic Health Qigong practice. From a neurophysiological point of view, frontal theta power has been found to increase with working memory load (Gevins et al., 1997; Jensen and Tesche, 2002; Onton et al., 2005). Challenging working memory may lead to a loss of a merely executive action control due to limited resource capacity. As a consequence, practitioners' attention is drawn away from cognitive engagement in everyday thoughts by a demanding movement control process in dynamic Health Qigong training (see Schmalzl et al., 2014). Further, a loss of cognitive action control toward a state of non-focusing and non-involvement on the everyday mind flow is one of the main aims in Eastern meditation techniques (for an overview see Tomasino et al., 2014). Especially in Buddhism-related meditation traditions a mindfulness state is reached by sustained attention on the body. Activations in midline fronto-central lobe structures associated with attentional processes possibly confirming the fundamental role of mindfulness shared by many Buddhist meditations (for an overview see Tomasino et al., 2014). Transferring these findings on the results obtained in the current study, we infer that different cognitive and attentional processes lead to the obtained temporal courses in EEG brain activity in *Wu Qin Xi* and *Liu Zi Jue*. While the state of internalized attention in *Liu Zi Jue* is reached by directing the focus of attentional processing internally due to binding of breathing capacities in *Liu Zi Jue*, a psychophysiological state of relaxation is reached delayed in *Wu Qin Xi* after 30 min. Possibly, executive control processes of the complex movement configurations rather than by breathing behavior mediated internalized attention play the main part mirrored in reduced alpha-1 and alpha-2 activity after 15 min. Thus, directing attention externally toward the movement, might lead to a more superficial movement execution without focusing breathing and on bodily sensations, which is mirrored in decreases in theta and alpha power.

Finally, movement configurations in *Liu Zi Jue* are reported from practitioners to be less complex than in *Wu Qin Xi*. Therefore, practitioners possibly have more attentional capacities to spend effort on control of breathing in *Liu Zi Jue* than on control of complex movement configurations as in *Wu Qin Xi*. Following this line of argumentation, the obtained results of increased theta, alpha-1 and alpha-2 activity can be interpreted in this manner.

To date, there are no systematical studies reported on the effects of *Liu Zi Jue* Qigong training on EEG brain activity. Recently, it was shown that *Liu Zi Jue* training made beneficial effects on pain consciousness and depression of elderly single women (Dong and Lee, 2013). As depression is correlated with reduced EEG alpha activity (see Başar et al., 2012), the present study gives insight into the underlying neurophysiological processes that mediate the beneficial effects of *Liu Zi Jue* in depression. *Liu Zi Jue* training increases low-frequency EEG (theta and alpha-1 activity) that regulate the brain activity of reduced alpha activity in depression patients down to the level of healthy subjects.

To our knowledge, the present study is the first one that compares general acute effects and the temporal course of dynamic Qigong *Wu Qin Xi* and *Liu Zi Jue* training on EEG brain activity. Summarizing, a relaxation effect in *Wu Qin Xi* and *Liu Zi Jue* in a sense of an evidence-based approach is to be stated: the dynamic Qigong techniques *Wu Qin Xi* and *Liu Zi Jue* induce increased midline fronto-central theta and shifts of alpha activity from posterior to anterior regions after 30 min of training. Thus, we obtained comparable patterns and intensities of EEG brain activity after 30 min in both Qigong techniques. Different temporal courses in EEG theta, alpha-1, and alpha-2 activity were demonstrated. In *Liu Zi Jue*, a continuous increase in EEG fronto-central theta, posterior alpha-1, and alpha-2 activity was obtained, whereas in *Wu Qin Xi* decreases of central and parietal alpha-1, and central alpha-2 activity was demonstrated after 15 min, followed by an increase after 30 min.

We argue that the differences regarding the temporal course of EEG brain activity result from a tighter control of breathing behavior during sound production in *Liu Zi Jue*. Further, we hypothesize that the control of performance of complex movement sequences in *Wu Qin Xi* leads to the observed pattern of EEG brain activity.

The results of our study have important implications for the design of interventions applying the dynamic Qigong techniques *Wu Qin Xi* and *Liu Zi Jue* especially with the indication for a strengthening of mental health and stress reduction. Especially in clinical populations who display reduced spontaneous alpha activity as in stress mediated diseases like burnout (van Luitjelaar et al., 2010; Tukaiev et al., 2012), but as well as in anxiety, depression, and bipolar disorders (Başar et al., 2012) a strong induction of alpha activity by Qigong practice is essential for the therapeutic success of the intervention. The data on the subjective psychophysiological state underline the reducing effect of both dynamic Qigong techniques on signs of tension, anxiety, and fatigue. Therefore, it is recommended, to practice *Wu Qin Xi* for 30 min to reach a psychophysiological effect of relaxation and attentiveness. In *Liu Zi Jue* a practice session of at least 15 min is recommended. Further research is needed to clarify the role of long-term training effects in both Qigong techniques on the temporal course of EEG brain activity. One further interesting question is whether the same effects and temporal courses in brain activity would be expected in clinical populations that show reduced alpha oscillations at resting baseline.

## Author contributions

The authors DH and WS cooperated on developing the theoretical framework and preparing the manuscript.

### Conflict of interest statement

The authors declare that the research was conducted in the absence of any commercial or financial relationships that could be construed as a potential conflict of interest.
